# A Great Industry

**Published:** 1875-10

**Authors:** 


### A GREAT AMERICAN INDUSTRY.

Twenty years ago we would have been deemed presumptuous if we had predicted, that, within a comparatively brief period, America might safely challenge France as a producer of silk goods. That this is literally true to-day, so far as the quality of American silks is concerned, we have abundant testimony. In the matter of quantity, we are, as yet, of course, far behind France. Still, our woven silk product this year will by no means be insignificant. The new industry is thriving abundantly, and is destined to reach a leading position among our manufactures.

What concerns us and the public most is, that the standard of quality shall be maintained; that our goods shall not only *equal* those of the best European looms, but shall, if possible, excell them.

We have been looking into the subject for our own information, and for the benefit of our readers, with especial reference to the quality of American silks, and are prepared to speak somewhat authoritatively upon this point.

We take the products of the leading silk-manufacturers, Messrs. Cheney Brothers, whose great factories are at Hartford and South Manchester, Connecticut, and whose warerooms are at 477 Broome Street, New York. Tested by any standard of comparison, the goods from this house compare favorably with those from the best-known makers in the old world. Examined side by side with the silks of Bonet, or Radsmier, or any of those old houses whose fame as silk-fabricateurs has long been world-wide, we find no cause for diminished pride in the quality of the American goods.

There is a solid, warm, costly, elegant appearance to the silks produced by the Messrs Cheney, which is lacking in nearly all the foreign products. They have all the genuine sheen of pure silk, but they have none of the meretricious, tinsel-like glitter, which is imparted by chemical agencies. They are without brilliant gloss, because no varnish has been added to the goods to give them an unnatural lustre, an added weight, an increased body. They are simply honest, natural silk goods, without adulteration or sophistication. Their weight is the weight of pure silk contained in the goods, and not the weight of metallic pigments used in conferring upon them a false bulk and a seeming excellence.

This matter of giving to silk goods an apparant quality which they are innocent of, is widely practised abroad. The loosely woven fabric is stuffed to its utmost capacity with metallic substances which impart stiffness and weight to the goods, while adding no atom of value. In fact the processes employed still further weaken a structure originally bad. From all such false pretences, the silks of the Cheneys are happily free.

A point which we have already mentioned, we would like to enforce here. We are sure it will appeal to the good sense of our regular readers. We allude to the total absence of garish display in these American silks. There is a class who prefer glitter, even though it is artificial. These are not our kind of people; they do not read our Journal except by accident, and then it does not make them happy. The extra gloss on so many French silks is *put on.* The Messrs. Cheney could impart the same tinseled aspect to their goods, but they will not do it. They prefer to do what in them lies to cultivate a taste in their fellow country-women, for soft, semi-lustrous, elegant, durable silk fabrics, to the exclusion of the cambric-looking affairs in which women of the "loud" type delight to revel.

In the matter of economy, we have satisfied ourselves that the choice is largely in favor of the Cheney silks. That they surpass in honest weight, in perfection of fabric—by which we mean freedom from bad threads and irregular places—and in durability—and by durability, we mean not only wearing power, but the power to wear long and well without presenting a glazed and worn aspect —we have secured abundant testimony.

A good silk from these looms, of whatever color, may be re-made as styles change,—may be reversed and turned, and will still look fresh. In the dark colors, if soiled it may be sponged with soap and water, and its natural elegance will not be diminished. Being a pure and natural product originally, without the harsh and wiry characteristics of the foreign goods, no ill-treatment is able to impart these unnatural qualities. The fabric may be worn out in time, of course; but, as one lady said, "it will look well as long as a thread remains."

This is because the absence of all foreign substances, the lack of the "weighting" process, of which we have spoken, and of all other modes of falsification, secure lasting elegance and great durability. Impure, "weighted," and other falsified silks "cut," and are soon disfigured and ruined. The goods of which we speak are warranted unchangeable in color, and they are clearly far more valuable than any foreign silks to be had at the same price. We wish every American woman who wears silks would for once try one of these made by the Messrs. Cheney. One trial would, we feel sure, make her a warmer advocate for these goods than we can ever be.

